# Shared correlates of prescription drug misuse and severe suicide ideation among clinical patients at risk for suicide

**DOI:** 10.1111/sltb.12685

**Published:** 2020-08-28

**Authors:** Joseph E. Logan, Allison M. Ertl, Whitney L. Rostad, Jeffrey H. Herbst, E. Ashby Plant

**Affiliations:** ^1^ Centers for Disease Control and Prevention (CDC) Division of Violence Prevention National Center for Injury Prevention and Control 4770 Buford Hwy NE Atlanta Georgia 30341 USA; ^2^ Department of Psychology Florida State University 1107 W. Call Street Tallahassee Florida 32306 USA

## Abstract

**Objective:**

Unintentional drug overdose and suicide have emerged as public health problems. Prescription drug misuse can elevate risk of overdose. Severe suicidal ideation increases risk of suicide. We identified shared correlates of both risk factors to inform cross‐cutting prevention efforts.

**Methods:**

We conducted a cross‐sectional study using the Military Suicide Research Consortium's Common Data Elements survey; 2012–2017 baseline data collected from 10 research sites were analyzed. The sample included 3962 clinical patients at risk of suicide. Factors examined in relation to the outcomes, prescription drug misuse and severe suicidal ideation, included demographic characteristics and symptoms of: hopelessness; anxiety; post‐traumatic stress disorder; alcohol use; other substance use; prior head/neck injury; insomnia; and belongingness. Poisson regression models with robust estimates provided adjusted prevalence ratios (aPRs) and 97.5% confidence intervals (CIs).

**Results:**

Medium and high (vs. low) levels of insomnia were positively associated with prescription drug misuse (aPRs *p* < 0.025). Medium (vs. low) level of insomnia was positively associated with severe suicidal ideation (aPR: 1.09; CI: 1.01–1.18). Medium and high (vs. low) levels of perceived belongingness were inversely associated with both outcomes (aPRs *p* < 0.025).

**Conclusions:**

Research should evaluate whether addressing sleep problems and improving belongingness can reduce prescription drug misuse and suicidal ideation simultaneously.

## 1 INTRODUCTION

In the past decade, rates of drug overdose deaths—mainly unintentional overdoses—and suicide have increased in the United States (Hedegaard, Curtin, & Warner, 2020; Hedegaard, Minino, & Warner, 2018; Rudd, Seth, David, & Scholl, 2016). The age‐adjusted drug overdose mortality rate increased over 200% between 1999 and 2017 from 6.1 to 21.7 deaths per 100,000 U.S. population, and 87% of the drug overdoses in 2017 were classified as unintentional overdoses (Hedegaard et al., 2018). In the same period, the age‐adjusted suicide rate increased 35%, from 10.5 to 14.2 deaths per 100,000 U.S. population (Hedegaard et al., 2020). The antecedents of unintentional drug overdose death and suicide may be similar even though firearms are the most common mechanism used in suicides (Jack et al., 2018; Sheu, Chen, & Hedegaard, 2015). Both heavily burden a similar demographic group (i.e., adult men of non‐Hispanic white race/ethnicity) (CDC WISQARS, 2020; Hedegaard et al., 2020; Rudd et al., 2016; Scholl, Seth, Kariisa, Wilson, & Baldwin, 2018) and could occur as maladaptive ways of coping with depression and physical pain (Grattan, Sullivan, Saunders, Campbell, & Von Korff, 2012; Han, Compton, Blanco, & Jones, 2018; Joiner, 2007; Petrosky et al., 2018; Rockett et al., 2014). Nevertheless, these public health problems are largely studied independently. Breaking silos of research to discover shared risk factors for unintentional drug overdose and suicide may inform strategies to prevent these public health problems simultaneously.

Identifying shared risks for fatal drug overdose and suicidal behavior is challenging, because decedents cannot share their precipitating risk exposures. Researchers have examined risk factors, or at least the circumstances of death, among those who died of suicide and other forms of injury through qualitative analyses of death scene investigation reports (e.g., coroner/medical examiner reports or law enforcement reports) (Logan, Ertl, & Bossarte, 2019; Logan, Hall, & Karch, 2011; Logan, Skopp, Karch, Reger, & Gahm, 2012) or, similarly, through psychological autopsy methods or medical record reviews (Nock et al., 2017). An alternative approach is to study outcomes (e.g., prescription drug misuse and severe suicidal ideation) that pose as key risk factors for the fatal behavior among a living sample. Taking more than prescribed quantities of drugs, particularly opioids, anti‐anxiety medications, and sedatives (e.g., benzodiazepines) can increase risk of overdose (Ballantyne, 2012; Jones & McAninch, 2015), or use of drugs commonly involved in overdose deaths such as heroin (Jones, Logan, Gladden, & Bohm, 2015). Similarly, severe levels of suicidal ideation that involve suicidal thoughts, lack of control over thoughts, suicidal impulses, and suicide plans can increase risk of suicidal behavior (Rogers & Joiner, 2018). Examining shared correlates of prescription drug misuse and severe suicidal ideation among a living sample may inform prevention strategies to reduce risk of death. Persons who misuse prescription drugs and attempt suicide often have other comorbid conditions related to pain and/or mental health (Bohnert, Ilgen, Louzon, McCarthy, & Katz, 2017; Garland, Riquino, Priddy, & Bryan, 2017; Han et al., 2018). However, examinations of symptoms associated with conditions such as prior head/neck injury, post‐traumatic stress disorder (PTSD), insomnia, and anxiety related to prescription drug misuse and severe suicidal ideation are still needed. These conditions are positively associated with suicide (Bryan & Clemans, 2013; Calabrese et al., 2011; Dobscha et al., 2014; Harnod, Harnod, Lin, Shen, & Kao, 2019; Ribeiro et al., 2012; Vanderploeg et al., 2015; Wisco et al., 2014), and persons with these symptoms are often prescribed drugs such as benzodiazepines (National Institute of Drug Abuse, 2018), which are susceptible to misuse (Blanco, Han, Jones, Johnson, & Compton, 2018).

In addition, there may be avenues to enhance primary prevention efforts. For example, literature on suicide prevention repeatedly shows that enhancing a sense of “belongingness” (i.e., having strong connections to family members, peers, and community) can serve a protective function against suicide (Bryan & Heron, 2015; Bryan, McNaughton‐Cassill, & Osman, 2013; Joiner, 2007; Kaminski et al., 2010; Mitchell, Gallaway, Millikan, & Bell, 2012). A sense of belongingness reduces feelings of isolation and gives a sense of confidence that someone will listen when seeking help for mental or emotional distress. Belongingness may also protect against prescription drug misuse. Prior research shows that loneliness can elevate risk of alcohol misuse (Akerlind & Hornquist, 1992; Schonfeld, Rohrer, Dupree, & Thomas, 1989) and smoking among adolescents (Seo & Huang, 2012). However, research on whether belongingness is inversely associated with prescription drug misuse needs exploration.

This study examines health‐related conditions and belongingness in relation to prescription drug misuse and severe suicidal ideation in a large clinical sample of civilians, Military Service Members, and Veterans at risk for suicide.

## 2 METHODS

### 2.1 Data source and population

The Military Suicide Research Consortium's (MSRC) Common Data Elements (CDE) survey data were used. This survey was developed by the directors of the MSRC and experts from the field of suicide research (Ringer et al., 2018). It was administered to 6555 participants at 26 MSRC‐funded research sites evaluating suicide prevention strategies.

For this analysis, we included 10 sites that sampled clinical patients with histories of suicidal ideation and behavior or non‐suicidal self‐directed violence. Patients were either currently in inpatient or outpatient clinical settings, referred to the study by a provider, or recruited based on recent self‐injury or a nonfatal suicide attempt. Depending on the research site, the survey was completed on paper, by interview, online, or using a combination of these approaches, and response rates for these 10 sites ranged from 86% to 100%. Sites that recruited from the general population in community settings were excluded. Also excluded were sites that recruited patients with specific conditions (e.g., anxiety sensitivity and major depressive disorder) that may bias the associations between risk factors ascertained and outcomes. A total sample of 3962 survey participants was included.

Details on patient inclusion/exclusion criteria and survey response rates for the 10 selected MSRC sites are provided online (Table S1). This study examined survey data collected at baseline *prior* to intervention exposure. Study sites were initiated from 2012 to 2017, distributed nationwide, and included civilians, Military Service Members, and Veterans (Ringer et al., 2018).

MSRC studies were funded through a competitive application process. Military Service Members represented all branches. Institutional Review Board approvals were obtained. Details on the population and MSRC CDEs can be accessed at https://msrc.fsu.edu/members/request‐access‐msrc‐database (Military Suicide Research Consortium).

CDEs are composed of 57 items on participant demographics, suicide‐related outcomes (e.g., ideation, plans, attempts), non‐suicidal self‐directed violent behaviors, and symptoms of psychiatric conditions, prior head/neck injury, substance use, and insomnia. The survey also collected responses on perceived belongingness. Survey questions were either created specifically for this survey (10 items) or drawn from existing, validated measures (47 items) (Ringer et al., 2018). Abbreviated forms of full scales capturing symptoms of the constructs were based on factor loadings from past research and content coverage (Ringer et al., 2018). Brief survey measures were compared to full scales, and all CDE shortened measures were correlated with the corresponding full measures with moderate to strong effect sizes (Ringer et al., 2018).

### 2.2 Variables

Outcome variables were “any prescription drug misuse” and “severe suicidal ideation.” Any prescription drug misuse was defined as reporting at least some misuse to the question “How often do you use prescription drugs more often or at greater quantities than prescribed?” Responses of some misuse ranged from “monthly or less” to “four or more times per week.” Given this study sample included those at risk of suicidal ideation and behavior, we examined severe suicidal ideation for the second outcome. Severe suicidal ideation was based on four items from the Depressive Symptom Inventory Suicidality Subscale (Metalsky & Joiner, 1997). Items capture within the past two weeks of taking the survey the degree of having thoughts of killing oneself, formulating plans to kill oneself, having little control over suicidal thoughts, and having impulses to kill oneself in response to (some/most/all) situations. For each item, responses were on a scale (0–3) with higher values reflecting greater severity. For each participant, the mean scale score across all items was calculated. Means were divided into equal thirds as closely as possible for the *entire* MSRC study population. Those with mean scores closest to the top 33rd percentile of the study population were considered to have high scores on suicidal ideation. Because this study focused on a high‐risk clinical subsample of the entire MSRC study population, the proportion of those considered to have high mean scores was skewed to 45.3%. This high score was deemed to be “severe” suicidal ideation for this study, because it equated to (on average) having at least some presence of each of the four suicidal ideation elements (i.e., thoughts, plan formulation, little control over thoughts, and impulsive thoughts) within two weeks of the survey.

Correlates in this study included participant demographic characteristics; symptoms of mental health (MH) factors (i.e., hopelessness, anxiety, and PTSD); frequency and intensity of alcohol use; other substance use (i.e., cocaine, heroin, methamphetamine [meth], marijuana, other pills (i.e., other than those prescribed)); current/recent insomnia symptoms; and prior symptoms of head/neck injury. One protective factor, perceived belongingness, was also examined. Demographic characteristics included age, sex, race/ethnicity, education (highest degree), and military status (i.e., currently serving, Veteran, and never served in the military [a.k.a., “civilians”]). Symptoms of MH factors, other substance use, and other health and relationship factors were provided from established scales (see online material; Table S2). Abbreviated versions of the full scales were only available for our sample, except for survey items capturing other substance use and head/neck injury. Because full scales were not used for some factors, we were precluded from using the official clinical scoring methods and thresholds. Alternatively, mean scores for each factor were calculated for the entire MSRC study population. Based on the distribution of the mean scores, the population was divided roughly into thirds similar to the suicidal ideation outcome variable (i.e., top 33% were considered high scores, middle 33% were considered medium scores, and so forth). This approach was used in other studies (Kaminski et al., 2010; Logan, Crosby, & Hamburger, 2011), and the abbreviated scales used in this study were cross‐validated with the full scales (Ringer et al., 2018). Other substance use and prior symptoms of head/neck injury were coded differently. Other substance use was divided into three groups (i.e., never used these drugs, used drugs monthly or less to 2–4 times per month, and used drugs 2 or more times per week). To identify prior symptoms of head/neck injury, the Traumatic Brain Injury‐4 (TBI‐4) screen was coded according to one of the criterion used by Olson‐Madden and colleagues (i.e., [ever/never] where an “ever” status was coded if the respondent answered “yes” to any of the four questions that asked about history of head‐/neck‐related injuries (Table S2) (Olson‐Madden et al., 2014). This coding approach for the TBI‐4 was compared to the Ohio State University TBI‐Identification method, a structured interview for identifying lifetime history of TBI; results yielded 74% sensitivity and 56% specificity (Brenner et al., 2013). Because of the potential for falsely identifying TBI history, this variable was referred to as symptoms of prior head/neck injury. More details on all variable definitions and scale items are provided in the online material (Table S2).

### 2.3 Analysis

The population was first described with respect to all variables. Poisson regression with robust estimates was used to estimate adjusted prevalence ratios (aPRs) for each outcome, accounting for all variables listed in the tables and clustering at study sites. Two models were tested, one for each outcome. Both models included the same variables with one exception. Prior research showed both outcomes are correlated (Ashrafioun, Bishop, Conner, & Pigeon, 2017; Guo et al., 2016; Schepis, Simoni‐Wastila, & McCabe, 2018) so prescription drug misuse was included in the model predicting severe suicidal ideation and vice versa. As a correlate, prescription drug misuse was entered in the model for suicidal ideation as a trinary variable: never (referent); one time per month (or less) to 2–4 times per month; and at least twice per week. Suicidal ideation was also entered into the model for prescription drug misuse as a trinary variable: low mean score (referent); medium mean score; and high or “severe” mean score. We used 97.5% confidence intervals (CIs) to minimize type 1 errors. Regression models only include those with known outcome data. For the model predicting any prescription drug misuse, 3873 respondents (97.8%) had known outcome data and were included; for severe suicidal ideation, 3897 respondents (98.4%) were included. All analyses were conducted with STATA version 12 (College Station, TX).

## 3 RESULTS

Study sample characteristics are provided in Table 1. Respondents were mostly aged 18–34 years (70.7%), male (61.8%), non‐Hispanic white (60.2%), and had at least some college education (61.8%). A high proportion (42.5%) of the sample included Military Service Members, 23.3% were Veterans, 25.6% were civilians, and 8.6% had unknown Military service. Eight‐hundred and eighty‐six (22.4%) respondents reported current misuse of prescription drugs, with 193 (4.9%) reporting misuse at least twice per week. Further, 1796 (45.3%) respondents had high scores on suicidal ideation (hereafter, “severe suicidal ideation”).

**Table 1:** Characteristics of study population: Military Suicide Research Consortium (MSRC) funded studies, 2012 to 2017

Characteristic	Total Participants (No. (%))
Age groups (years)	
18–34	2801 (70.7)
35–54	749 (18.9)
55–74	343 (8.7)
75+	9 (0.2)
Unknown	60 (1.5)
Gender	
Male	2447 (61.8)
Female	1384 (34.9)
Transgender/other	44 (1.1)
Unknown	87 (2.2)
Race/Ethnicity	
Non‐Hispanic white	2383 (60.2)
Non‐Hispanic black	496 (12.5)
Non‐Hispanic American Indian/Alaskan Native	35 (0.9)
Non‐Hispanic Asian/Pacific Islander	122 (3.1)
Non‐Hispanic multiracial	34 (0.9)
Other non‐Hispanic race	160 (4.0)
Hispanic ethnicity	399 (10.1)
Other ethnicity	66 (1.7)
Unknown	267 (6.7)
Education (highest degree)	
Less than high school	150 (3.8)
High school diploma/general equivalency diploma	1275 (32.2)
Some college	1784 (45.0)
College degree	493 (12.4)
Beyond college degree	175 (4.4)
Unknown	85 (2.2)
Military service	
Currently serving	1685 (42.5)
Veteran	922 (23.3)
No military service (Civilians)	1013 (25.6)
Unknown	342 (8.6)
Prescription drug misuse	
Never	2987 (75.4)
One time per month (or less) to four times per month	693 (17.5)
At least twice per week	193 (4.9)
Unknown	89 (2.3)
Suicidal ideation^a^	
Low score	771 (19.5)
Medium score	1330 (33.6)
High score (Severe)	1796 (45.3)
Unknown	65 (1.6)
Total	3962 (100.0)

^a^ Based on four items from the Depressive Symptom Inventory Suicidality Subscale: thoughts of killing oneself, formulating plans to kill oneself, having little control over suicidal thoughts, and having impulses to kill oneself in response to (some/most/all) situations.

In the adjusted analysis, no individual demographic characteristic was significantly associated with both outcomes (Table 2). Having a medium or higher (vs. low) score of insomnia symptoms was positively associated with prescription drug misuse; a medium (versus low) score of insomnia symptoms was positively associated with severe suicidal ideation (aPR: 1.09; CI: 1.01–1.18). Medium and high scores of perceived belongingness were inversely associated with any prescription drug misuse and severe suicidal ideation (all aPRs *p* < 0.025) (Table 3).

**Table 2:** Demographic correlates of prescription drug misuse and severe suicidal ideation: Military Suicide Research Consortium (MSRC) Funded Studies, 2012 to 2017

Characteristic	Any prescription drug misuse		Severe suicidal ideation	
	No (%)	Adjusted prevalence ratio (97.5% CI)^a^, ^b^	No (%)	Adjusted prevalence ratio (97.5% CI)^a^, ^c^
Age groups (years)				
18–34	557 (20.2)	Referent	1275 (46.0)	Referent
35–54	212 (28.8)	1.21 (1.15–1.28)	351 (47.8)	0.89 (0.79–1.00)
55–74	100 (30.9)	1.47 (1.28–1.70)	137 (41.9)	0.80 (0.65–0.99)
75+	* (*)	*	* (*)	*
Gender				
Male	469 (19.6)	Referent	1090 (45.2)	Referent
Female	388 (28.6)	1.12 (1.00–1.24)	635 (46.5)	0.92 (0.81–1.04)
Transgender/other	9 (21.4)	0.91 (0.75–1.09)	25 (59.5)	1.34 (1.12–1.61)
Race/Ethnicity				
Non‐Hispanic white	608 (25.9)	Referent	1137 (48.3)	Referent
Non‐Hispanic black	88 (18.1)	0.77 (0.56–1.05)	207 (42.1)	0.88 (0.77–1.01)
Non‐Hispanic American Indian/Alaskan Native	10 (29.4)	1.13 (0.78–1.64)	16 (45.7)	0.91 (0.68–1.21)
Non‐Hispanic Asian/Pacific Islander	35 (29.2)	1.17 (0.96–1.44)	59 (48.8)	1.01 (0.92–1.10)
Non‐Hispanic multiracial	10 (29.4)	0.85 (0.61–1.17)	22 (64.7)	1.06 (0.83–1.35)
Other Non‐Hispanic race	30 (19.1)	0.92 (0.79–1.09)	65 (41.1)	0.85 (0.63–1.15)
Hispanic ethnicity	63 (16.0)	0.91 (0.73–1.14)	164 (41.4)	0.91 (0.73–1.13)
Other ethnicity	* (*)	*	20 (30.3)	0.75 (0.43–1.32)
Education (highest degree)				
Less than high school	35 (24.0)	Referent	74 (50.3)	Referent
High school diploma or equivalency diploma	206 (16.5)	1.18 (0.81–1.72)	530 (42.0)	1.00 (0.88–1.13)
Some college	424 (24.1)	1.23 (0.83–1.82)	847 (48.0)	1.07 (0.95–1.20)
College degree	150 (30.8)	1.34 (0.75–2.38)	232 (47.5)	1.04 (0.82–1.32)
Beyond college degree	54 (31.4)	1.54 (0.93–2.54)	80 (46.5)	1.15 (0.80–1.66)
Military service				
Currently serving	165 (10.0)	0.47 (0.38–0.58)	675 (40.3)	0.95 (0.51–1.74)
Veteran	258 (29.2)	0.72 (0.58–0.89)	435 (49.0)	1.08 (0.85–1.38)
No military service (civilians)	328 (32.9)	Referent	506 (50.8)	Referent

^a^ All models adjust for age, gender, race/ethnicity, education, military service, and all other characteristics listed in table 3. Models also accounted for unknown values of each variable; unknown values were not displayed.

^b^ Sample is among those with known information on prescription drug misuse (*N* = 3873).

^c^ Sample is among those with known information on suicidal ideation (*N* = 3897).

* Value suppressed because of low counts.

**Table 3:** Health‐related correlates of prescription drug misuse and severe suicidal ideation: Military Suicide Research Consortium (MSRC) Funded Studies, 2012 to 2017

Symptoms of:	Any prescription drug misuse		Severe suicidal ideation	
	No (%)	Adjusted prevalence ratio (97.5% CI)^a^, ^b^	No (%)	Adjusted prevalence ratio (97.5% CI)^a^, ^c^
Mental health (MH) factors, Symptoms of:				
Suicidal ideation				
Low score	99 (12.9)	Referent		
Medium score	296 (22.4)	1.08 (0.92–1.27)		
High score	490 (27.5)	1.22 (1.01–1.48)		
Hopelessness				
Low score	249 (25.9)	Referent	394 (40.7)	Referent
Medium score	273 (20.5)	0.94 (0.74–1.20)	559 (41.9)	1.08 (0.82–1.41)
High score	362 (23.1)	0.99 (0.82–1.21)	835 (53.0)	1.20 (0.84–1.72)
Anxiety				
Low score	231 (17.6)	Referent	463 (35.1)	Referent
Medium score	384 (24.3)	1.03 (0.96–1.11)	744 (47.0)	1.16 (1.02–1.31)
High score	270 (27.8)	1.02 (0.92–1.13)	585 (60.0)	1.40 (1.20–1.62)
Post‐traumatic stress disorder				
Low score	131 (13.0)	Referent	357 (35.2)	Referent
Medium score	322 (23.2)	1.27 (1.06–1.51)	619 (44.5)	1.07 (0.90–1.27)
High score	344 (28.8)	1.30 (1.18–1.43)	653 (54.5)	1.15 (0.92–1.43)
Substance use				
Alcohol use				
Low score	226 (16.0)	Referent	644 (44.2)	Referent
Medium score	307 (24.5)	1.34 (1.10–1.63)	559 (44.5)	0.98 (0.91–1.04)
High score	352 (30.3)	1.54 (1.33–1.79)	585 (50.2)	1.07 (0.99–1.16)
Other substance use				
Never	453 (15.6)	Referent	1272 (43.8)	Referent
One time per month (or less) to 2–4 times per month	194 (42.9)	1.88 (1.42–2.49)	230 (50.9)	0.98 (0.92–1.06)
At least twice per week	238 (47.0)	2.08 (1.44–2.99)	283 (55.8)	1.06 (0.95–1.19)
Prescription drug misuse				
Never			1294 (43.4)	Referent
One time per month (or less) to 2–4 times per month			370 (53.4)	1.08 (1.01–1.16)
At least twice per week			120 (62.5)	1.19 (1.00–1.43)
Other health factors and perceived belongingness				
Symptoms of prior head/neck injury				
Never	283 (17.8)	Referent	690 (43.3)	Referent
Ever	603 (26.5)	1.25 (1.09–1.44)	1098 (48.1)	1.00 (0.89–1.12)
Insomnia Symptoms				
Low score	149 (14.8)	Referent	363 (36.1)	Referent
Medium score	396 (25.3)	1.39 (1.16–1.66)	738 (46.9)	1.09 (1.01–1.18)
High score	341 (26.2)	1.42 (1.22–1.65)	691 (53.0)	1.10 (1.00–1.21)
Belongingness				
Low score	483 (28.3)	Referent	1000 (58.2)	Referent
Medium score	288 (20.6)	0.87 (0.79–0.97)	586 (41.7)	0.77 (0.68–0.87)
High score	115 (15.1)	0.86 (0.79–0.94)	206 (27.1)	0.57 (0.45–0.73)

Note:

Description of calculated variables are provided in Table S2.

^a^ All models adjust for age, gender, race/ethnicity, education, military service, and all other characteristics listed in table 3. Models also accounted for unknown values of each variable; unknown values were not displayed.

^b^ Sample is among those with known information on prescription drug misuse (*N* = 3873).

^c^ Sample is among those with known information on suicidal ideation (*N* = 3897).

Demographic factors positively associated with any prescription drug misuse included age (35–54 and 55–74 vs. 18–34 years; aPRs *p* < 0.025) and female (vs. male) gender (aPR: 1.12; CI: 1.00–1.24, *p* < 0.025) (Table 2). Compared to civilians, prescription drug misuse was less prevalent among currently serving Military Service Members (aPR: 0.47; CI: 0.38–0.58) and Veterans (aPR: 0.72; CI: 0.58–0.89) (Table 2). Further, prescription drug misuse was positively associated with having a medium and high (versus low) score of PTSD symptoms, a medium and high (vs. low) score of alcohol use, any other substance use (vs. never), and any prior symptoms of head/neck injury (vs. never) (all aPRs *p* < 0.025) (Table 3).

Severe suicidal ideation was less prevalent among those aged 55–74 years versus 18–34 years. Reporting transgender/other (vs. male) gender was positively associated with severe suicidal ideation (aPR: 1.34; CI: 1.12–1.61) (Table 2). Medium and high (vs. low) scores for anxiety symptoms were positively associated with severe suicidal ideation (all *p* < 0.025) (Table 3). Finally, severe suicidal ideation was positively correlated with prescription drug misuse (i.e., one time per month (or less) to 2–4 times per month) (aPR: 1.08; CI: 1.01–1.16) (Table 3).

## 4 DISCUSSION

This study examined shared health and relationship correlates for prescription drug misuse and severe suicidal ideation among a clinical sample at risk for suicide. Similar to another study (Bohnert et al., 2013), we found anxiety was more directly related to suicidal ideation, while alcohol and other substance use were stronger correlates of prescription drug misuse. Similar findings were reported when histories of those who died of intentional (suicide) overdose were compared to unintentional overdose among nearly 3.3 million Veterans Health Administration patients (Bohnert et al., 2013). MH‐related factors were more positively correlated with intentional overdose, and substance use factors were more associated with unintentional overdose (Bohnert et al., 2013).

We also found insomnia symptoms to be positively correlated with both outcomes, even after accounting for symptoms of PTSD, hopelessness, anxiety, prior head/neck injury, and substance use; all factors associated with the outcomes and sleep problems (Al‐Ameri, Mohsin, & Abdul Wahid, 2019; Blumenthal, Taylor, Cloutier, Baxley, & Lasslett, 2019; Chakravorty et al., 2013; Hansen et al., 2018). The association between insomnia symptoms and severe suicidal ideation may have occurred because they are both symptoms of depression (American Psychiatric Association, 2013). Insomnia can also be a common side effect of some drug treatments for anxiety and depression, such as selective serotonin‐reuptake inhibitors (Argyropoulos & Wilson, 2005; Wichniak, Wierzbicka, Walecka, & Jernajczyk, 2017). Benzodiazepines (e.g., clonazepam) are sometimes prescribed to treat such symptoms or side effects (Minkel & Krystal, 2013). While benzodiazepines are controlled prescription drugs, they are susceptible to misuse (Blanco et al., 2018), which may partially explain the link between insomnia and prescription drug misuse in this study. More work is needed to explore this connection. Based on our findings, insomnia or sleep management strategies such as cognitive behavioral therapy (CBT) should be evaluated on the reduction of insomnia and its potential effects on suicidal ideation and prescription drug misuse (Edinger & Means, 2005; Manber et al., 2011).

Perceived belongingness was inversely associated with both outcomes in this study. Previous research shows that strengthening belongingness can be associated with lowering suicide risk (Bryan & Heron, 2015; Bryan et al., 2013; Kelley et al., 2019), but our finding that belongingness is inversely associated with prescription drug misuse has not been heavily explored and may provide a new direction to proactively prevent prescription drug misuse before it begins. Enhancing belongingness may disrupt some of the forces that drive prescription drug misuse. For example, one theory attributes the causes of the increased rates of opioid overdose and suicide deaths to increased demand for prescription opioids (Bohnert & Ilgen, 2019), and suggest opioid use, like alcohol use, may be a coping device for declining hopefulness about future wealth and opportunities among the working classes (Bohnert & Ilgen, 2019; Case & Deaton, 2015; Dasgupta, Beletsky, & Ciccarone, 2018). Reducing isolation and enhancing connections with others who exhibit healthy prosocial behaviors may replace maladaptive coping devices, improve hopefulness, and strengthen judgment (Center for Substance Abuse Treatment, 1999). Future research is needed to further develop and test the efficacy of prevention interventions that enhance belongingness on lowering risk of both prescription drug misuse and suicidal ideation.

The positive association between symptoms of head injury and risk of suicide is established (Harnod et al., 2019), and similar to findings on the association of TBI with suicide risk (Madsen et al., 2018; Shura et al., 2018; Soberay, Hanson, Dwyer, Plant, & Gutierrez, 2018). However, we did not find a strong association between prior symptoms of head/neck injury and severe suicidal ideation in our adjusted analysis with a clinical sample at high risk for suicide, though these symptoms were positively associated with prescription drug misuse. Research on the relationship of head/neck injury to prescription drug misuse has not received much attention, but positive associations have been reported for TBI, especially among Military Service Members (Golub & Bennett, 2013). Prescription drug misuse among those reporting prior symptoms of head/neck injury may be related to coping with ongoing pain (Irvine & Clark, 2018) and symptoms of depression (Harnod et al., 2019). It may also be related to coping with some secondary symptoms such as difficulties with social interactions, memory, vocational skills, and work performance; these symptoms have been associated with TBI (Lundin, de Boussard, Edman, & Borg, 2006; Tyerman, 2012). In 2013, TBI was diagnosed in almost 2.8 million of the 26 million injury‐related emergency department visits, hospitalizations, and deaths in the United States (CDC, 2019), with many survivors living with TBI‐related disabilities (Lundin et al., 2006; Tyerman, 2012). Given the frequency of TBIs, and their association with head injuries, monitoring those with histories of TBIs for prescription drug misuse may be worthy of attention (Harnod et al., 2019).

Prescription drug misuse was an independent predictor of severe suicidal ideation in this sample. While this study was unable to examine the specific prescription drugs misused, other studies have found associations between prescription pain medication and suicidal behavior. O’Neill and colleagues (2019) showed a strong link between prescription pain medication and suicide among a population sample in Northern Ireland (O'Neill et al., 2019). Other studies showed higher doses of prescription opioid use are associated with suicide (Han et al., 2018; Ilgen et al., 2016). Ashrafioun and colleagues (2019) also discovered a similar finding that opioid use disorder along with depression or alcohol use disorder increased risk of suicide attempt among 226,444 Military Veterans seeking pain care (Ashrafioun et al., 2019). More research is needed to understand how prescription drug misuse and severe suicidal ideation are linked. At a minimum, prescription drug misuse might be another factor used to help assess suicide risk (Bohnert & Ilgen, 2019).

Our study is subjected to limitations. First, the study was not nationally representative and only included convenience samples of clinical patients at risk of suicide recruited into 10 research studies. If this study was conducted on a sample that reflected the general population, different shared correlates might have emerged. Second, the survey item capturing prescription drug misuse did not collect details on the prescription drugs (e.g., opioids, benzodiazepines, or another type) or whether the increased quantities used were the result of inadvertent nonadherence; such details could have helped characterize the nature of the drug misuse. It also did not incorporate whether drugs were prescribed by multiple providers or obtained illicitly. Only an estimated 35% of those who misuse prescription pain relievers use medications prescribed by one doctor (Substance Abuse & Mental Health Services Administration, 2019). Therefore, this measure most likely underestimated the scope of the prescription drug misuse problem. Relatedly, the “other substance use” variable captured other pill use, which may have involved prescription drug misuse related to drug diversion (i.e., obtaining prescription drugs illegally) and accounted for some of its correlation with prescription drug misuse. Fourth, the survey did not directly collect information on pain, which is a shared risk factor for both suicide (Garland et al., 2017) and prescription drug misuse (Han et al., 2018) as well as sleep problems (Finan, Goodin, & Smith, 2013), which could have at least partially explained the positive association identified between sleep problems and the outcomes. Fifth, we were also unable to examine a depression scale with the outcomes, which could also explain the associations between sleep problems and the outcomes. However, sleep problems remained positively associated with the outcomes after adjusting for depression symptoms like hopelessness and anxiety. Sixth, this self‐report survey could have been subject to social desirability bias. Last, this study is cross‐sectional in design, and therefore, we cannot infer causality in the observed associations.

Strategies that address risk for both prescription drug misuse and severe suicidal ideation may help reduce rising rates of drug overdoses and suicide. This study suggests the potential importance of managing sleep problems and enhancing belongingness as areas to explore for prevention. There are strategies that can enhance connectedness in a community setting that show promise with reducing suicidal behaviors already (Coppens et al., 2014; Kral et al., 2009; WHO, 2018), but more work is needed to evaluate such efforts with respect to reducing prescription drug misuse. Further, more studies are needed to rigorously evaluate therapies for sleep problems on prescription drug misuse and severe suicidal ideation. Future studies should also explore whether there are shared correlates of suicide or drug overdose death using different study methods such as psychological autopsy review, medical chart review, or qualitative analyses of coroner/medical examiner reports and more studies are needed to improve understanding of the causal pathways of our discovered associations.

## 5 DISCLAIMERS

None of the authors have a conflict of interest with this manuscript or the research. No financial disclosures were reported by the authors. The findings and conclusions in this manuscript are those of the authors and do not necessarily represent the official position of the Centers for Disease Control and Prevention. The data collection was supported in part by the Military Suicide Research Consortium (MSRC), an effort supported by the Office of the Assistant Secretary of Defense for Health Affairs under Award Nos. (W81XWH‐10‐2‐0181 and W81XWH‐10‐2‐0178). Opinions, interpretations, conclusions, and recommendations are those of the authors and are not necessarily endorsed by the MSRC or the Department of Defense.

## Supporting information

Tables S1‐S2
