# Supplementary material for: Shared correlates of prescription drug misuse and severe suicide ideation among clinical patients at risk for suicide
Source: Suicide Life Threat Behav. 2020 Aug 28;50(6):1276–87. doi: 10.1111/sltb.12685 (PMC7754473; doi:10.1111/sltb.12685)
Supplement: Supplementary file 1 — Tables S1‐S2 [file SLTB-50-1276-s001.docx]

**ONLINE MATERIAL^a^**

| **eTable 1: Characteristics of Samples Selected from the Military Suicide Research Consortium’s Funded Sites, 2012 to 2017** | | | | | | | | |
| --- | --- | --- | --- | --- | --- | --- | --- | --- |
| **Site code** | **N** | **CDE Response Rate** | **Sample** | **Setting** | **Patient status** | **Recruitment Method** | **Inclusion Criteria** | **Exclusion Criteria** |
| 2 | 406 | 86% | Veterans & civilians | Clinical | Inpatient, emergency | Providers informed all patients with a recent suicide attempt of the study. Interested individuals met with member of research team for more information. | 1) Aged 18 years or older; 2) seeking treatment after suicide attempt; 3) speak/read English | 1) Presence of any factor impeding ability to provide informed consent; 2. suicidal ideation without attempt |
| 3 | 176 | 98% | Veterans | Clinical | Inpatient | Admitted to inpatient care for suicidal behavior; Research team made aware of all patients admitted for suicidal behavior. After admission, potential participants informed of study | 1) Aged 18-89 years; 2) Veteran; 3) admitted to inpatient care for suicidal behavior | 1) Presence of any factor impeding ability to provide informed consent |
| 6 | 19 | 100% | Veterans | Clinical | Outpatient | Clinicians providing Dialectical Behavior Therapy who were in contact with patients at risk of suicide informed them of the study; interested individuals met with a member of research team for more information | 1) Aged 18 years or older; 2) Veteran (preferred those of Operations Enduring Freedom and Iraqi Freedom; 3) assessed as high risk for self-harm when entered program; 4) own and use Android phone; 5) identified as clinically suitable for program or treatment as usual (TAU); 6) speak/ read English | 1) Presence of any factor impeding ability to provide informed consent; 2) admitted to VA Medical Center inpatient psychiatric unit greater than 2 weeks during study |
| 7 | 105 | 99% | Veterans | Clinical | Outpatient | Clinicians in contact with at risk patients from multiple VA Medical Center programs referred patients to the study. Interested patients met with member of research team for more information. | 1) Aged 18 years or older; 2) Veteran (preferred women of Operations Enduring Freedom, Iraqi Freedom, and New Dawn); 3) determined at high risk for suicidal behavior; 4) receiving active treatment from VA Medical Center clinic; 4) own and use Android phone or iPhone; 5) identified as clinically suitable for program or TAU. | 1) Presence of any factor impeding ability to provide informed consent; 2) moderate or severe dementia/ cognitive disturbance; 3) documentation of terminal illness; 4) admitted to VA Medical Center inpatient psychiatric unit |
| 9 | 657 | 96% | Current Military | Clinical | Inpatient, outpatient, emergency | Providers inform all patients presenting for suicidal behavior of study. Interested patients met with a study-affiliated continuity clinician for more information. | 1) Aged 18 years or older; 2) Military personnel; 3) admission to hospital for suicidal behavior; 4) own and use phone that can receive text messages; 5) speak/ read English | 1) Presence of any factor impeding ability to provide informed consent; 2) intervention deemed as contraindicated by treating clinician (e.g., paranoia exacerbated by being contacted); 3) prisoner/ under judicial order (i.e., participation not truly voluntary) |
| 13 | 1044 | 97% | Current Military | Clinical | Inpatient, outpatient, emergency | Patients with elevated risk of suicide | Active duty or Veterans at elevated suicide risk. |  |
| 14 | 139 | 96% | Veterans | Clinical | Inpatient | Patients admitted for suicidal behavior were approached by member of research team and informed of study while on inpatient unit. | 1) Veteran; 2) inpatient admission for suicidal behavior; | 1) Psychotic symptoms and/or significant cognitive impairment |
| 20 | 48 | 98% | Veterans | Clinical | Outpatient | Veterans with suicide ideation. | 1) Veterans ages 18-89 years; 2) eligible or engaged in primary care, behavioral health laboratories, mental health clinics, or other relevant clinical program at the VA Medical Center; 3) can attend research sessions at the VA Medical Center; 4) agrees to participate in the week-long assessment portion of the study; 5) score of 4 or greater on the Suicide Behavior Questionnaire-Revised. | Inability to read English, respond to verbal questions vocally, or read and complete printed self-report measures |
| 25 | 1079 | 92% | Civilians & Veterans | Clinical | online | Recent and severe history of non-suicidal self-injury and/or nonfatal suicide attempts | 1) Aged 18 years or older; 2) English fluency; and 3) recent, frequent non-suicidal self-cutting (i.e., ≥2 self-cutting episodes in the last two weeks), a nonfatal suicide attempt in the past year, or recent, frequent active suicidal ideation (i.e., active ideation more days than not in the past two weeks) |  |
| 26 | 289 | 93% | Veterans | Clinical | Inpatient, outpatient | Admitted to a VA inpatient psychiatric unit, reported either thinking about suicide or engaging in suicidal behavior | 1) Aged 18-89 years; 2) endorsement of suicidal ideation and/or behavior upon admission to the inpatient psychiatric unit or within the first 72 hours following admission; 3) able to provide a phone number and the address of a residence at which they can be reached; 4) planned location of discharge is a safe environment for the provider to visit; 5) agree to receive the intervention (active site participants only); 6) ability to adequately respond to questions regarding the informed consent procedure | 1) Receiving services from a mental health Intensive case management or domiciliary program or being directly transferred to further inpatient or residential treatment; 2) enrolled in other intervention studies that may affect the outcome of this study, or where this study may affect the outcome of the other study, until the subject has completed their participation in the other study; 3) current involvement in the criminal justice system as a prisoner or ward of the state. |

| **eTable 2: Data Dictionary, Military Suicide Research Consortium’s Funded Sites** | | | |
| --- | --- | --- | --- |
| **Variable** | **Survey Questions and Responses** | **Timeframe** | **Calculated Variable Description** |
| **Prescription drug misuse** | How often do you use prescription drugs more often or at greater quantities than prescribed?  1) never  2) monthly or less  3) 2-4times/month  4) 2-3times/week  5) 4+ times/week  6) unknown | Current | For the binary outcome, this variable was partitioned into two levels: 1) never); and 2) at least some (monthly or less to at least twice per week);  Prescription drug misuse was also entered as a correlate in the model developed for the outcome “severe suicidal ideation.” To increase variability, this variable was entered with three levels, similar to the other substance use variable. The three levels included: 1) never; 2) at least monthly or less to 2-4 times per month; and 3) at least twice per week. The “unknown” value, category 6, was not included in calculations and “unknown” values were accounted for in the regression models. |
| **Suicidal ideation** | Items were from the Depressive Symptom Inventory Suicidality Subscale  Item 1  1) I do not have thoughts of killing myself.  2) Sometimes I have thoughts of killing myself.  3) Most of the time I have thoughts of killing myself.  4) I always have thoughts of killing myself.  Item 2  1) I am not having thoughts about suicide. 2) I am having thoughts about suicide but have not formulated any plans. 3) I am having thoughts about suicide and am considering possible ways of doing it. 4) I am having thoughts about suicide and have formulated a definite plan.  Item 3  1) I am not having thoughts about suicide. 2) I am having thoughts about suicide but have these thoughts completely under my control. 3) I am having thoughts about suicide but have these thoughts somewhat under my control. 4) I am having thoughts about suicide but have little or no control over these thoughts.  Item 4  1) I am not having impulses to kill myself. 2) In some situations I have impulses to kill myself. 3) In most situations I have impulses to kill myself. 4) In all situations I have impulses to kill myself. | Last two weeks | Variable was based on the mean score across items. Higher mean scores reflect greater symptom severity. The mean only incorporated the questions answered. An estimated 94.34% of the population answered all four questions; 1.65% answered three questions; 0.15% answered two questions; 0.08% answered one question; 3.78% answered none of the questions. The mean scores were grouped into one of three groups. The "lower third" had a mean score of 1.0; the "middle third" included those with a mean score of 1.0-2.0. The “upper third" included participants who had a mean score of 2.1-4.0. Those in the upper third were considered to have “severe suicidal ideation.” The binary outcome was divided between those in the upper third, or severe suicidal ideation” versus those in the bottom two-thirds.  Suicidal ideation was also entered as a predictive correlate for the model developed for the outcome “any prescription drug misuse.” As a correlate, suicidal ideation was entered with three levels, all calculated using the same method as above. |
| **Hopelessness** | Items were from the Beck Hopelessness Scale. Items were not displayed because of copyright related reasons. | Past week | Variable was based on the mean score across items. Two items were reverse coded so higher mean scores reflect more hopelessness. The mean is calculated as the total score across all items divided by the number of items answered. An estimated 92.25% of the population answered all questions; 0.81% answered two question; 0.46% answered one question; 6.45% did not answer any of the questions. The mean scores were grouped into one of three groups (i.e., "lower third," “middle third,” and upper third). Details on score thresholds had to be suppressed for copyright related reasons. |
| **Anxiety** | Items were from the Anxiety Sensitivity Index (Anxiety-All Cognitive Concerns dimension). Responses to each item include: 1) very little; 2) a little; 3) some; 4) much; and 5) very much  Item 1- When my thoughts seem to speed up, I worry that I might be going crazy.  Item 2- When my mind goes blank, I worry that there is something terribly wrong with me.  Item 3- When I feel “spacey” or spaced out, I worry that I may be mentally ill.  Item 4- When I have trouble thinking clearly, I worry that there is something wrong with me.  Item 5- When I cannot keep my mind on a task, I worry that I might be going crazy. | Current | Variable was based on the mean score across items. The mean is calculated as the total score across all items divided by the number of items answered. Higher mean scores reflect more anxiety. An estimated 82.29% of the population answered all questions; 0.38% answered four question; 0.06% answered three questions; 0.08% answered two questions; 0.18% answered only one question; 17.01% did not answer any of the questions. The mean scores were grouped into one of three groups.  The "lower third" had a mean score of 1.0-2.0; the "middle third" included those with a mean score of 2.0-3.75. The “upper third" included participants who had a mean score of 3.8-5.0. |
| **Post-traumatic stress disorder** | Items came from the Post-traumatic Stress Disorder Checklist. Responses included: 1) not at all; 2) a little bit; 3) moderately; 4) quite a bit; and 5) extremely  Item 1- Repeated, disturbing memories, thoughts, or images of a stressful military experience  Item 2- Repeated, disturbing dreams of a stressful military experience  Item 3- Suddenly acting or feeling as if a stressful military experience were happening again (as if you were reliving it)  Item 4- Having physical reactions (e.g., heart pounding, trouble breathing, sweating) when something reminded you of a stressful military experience  Item 5- Avoiding thinking about or talking about a stressful military experience or avoiding having feelings related to it  Item 6- Avoiding activities or situations because they reminded you of a stressful military experience  Item 7- Being “super alert” or watchful or on guard  Item 8- Feeling jumpy or easily startled | Past Month | Variable was based on the mean score across items. The mean is calculated as the total score across all items divided by the number of items answered. Higher mean scores reflect greater symptom severity. An estimated 85.48% of the population answered all questions; 1.80% answered seven question; 0.26% answered six question; 0.11% answered five question; 0.05% answered four question; 0.12% answered three questions; 0.18% answered two questions; 0.05% answered only one question; 11.96% did not answer any of the questions. The mean scores were grouped into one of three groups. The "lower third" had a mean score of 1.0-1.86; the "middle third" included those with a mean score of 1.87-3.43. The “upper third" included participants who had a mean score of 3.44-5.0). |
| **Alcohol use** | Items came from the Alcohol Use Disorder Identification Test. Questions and responses included:  Item 1- How often do you have a drink containing alcohol?  1) never  2) monthly or less  3) 2-4times/month  4) 2-3times/week  5) 4+ times/week  6) unknown  Item 2- How many standard drinks containing alcohol do you have on a typical day?  1) 0,or 1-2  2) 3-4  3) 5-6  4) 7-9  5) 10+  6) unknown  Item 3- How often do you have six or more drinks on one occasion?  1) never  2) less than monthly  3) monthly  4) weekly  5) daily/almost daily  6) unknown | Current | Variable was based on the mean score across items. The “unknown” values, category 6 in each item, were not included in calculations and “unknown” values were accounted for in the regression models. The mean was calculated as the total score across all items divided by the number of items answered. Higher mean scores reflect greater alcohol use/misuse. An estimated 84.71% of the population answered all questions; 8.54% answered two questions; 0.37% answered only one question; 6.38% did not answer any of the questions. The mean scores were grouped into one of three groups. The "lower third" had a mean score of 1.0-1.33; the "middle third" included those with a mean score of 1.34-2.33. The “upper third" included participants who had a mean score of 2.34-5.0). |
| **Other substance use** | How often do you use other substances (e.g., marijuana, cocaine, heroin, meth, pills, etc.)?  1) never  2) monthly or less  3) 2-4times/month  4) 2-3times/week  5) 4+ times/week  6) unknown | Current | This variable was entered with three levels, similar to the prescription drug misuse variable. The three levels included: 1) never; 2) at least monthly or less to 2-4 times per month; and 3) at least twice per week. The “unknown” value, category 6, was not included in calculations and “unknown” values were accounted for in the regression models. |
| **Prior head/neck injury** | Symptoms of prior head/neck injury. Responses included: 1) no and 2) yes.  Item 1- Have you ever been hospitalized or treated in an emergency room following a head or neck injury?  Item 2- Have you ever been knocked out or unconscious following an accident or injury?  Item 3- Have you ever injured your head or neck in a car accident or from some other moving vehicle accident?  Item 4- Have you ever injured your head or neck in a fight or a fall? | Ever | This variable was dichotomized as ever/never. An individual received “ever” status if he/she answered “yes” to any of the four items. An individual received a “never” status if the individual answered “no” to all of the items. Among this sample, 412 (6.3%) respondents did not answer any questions; they received an “unknown” status. 46 (0.7%) respondents had answered “no” to some items but did not respond to others items. These individuals were categorized as “unknown” as well. |
| **Insomnia** | Items came from the Insomnia Severity Scale. Items and Responses include:  Item 1- Difficulty falling asleep  1) none  2) mild  3) moderate  4) severe  5) very severe  6) unknown  Item 2- Difficulty staying asleep  1) none  2) mild  3) moderate  4) severe  5) very severe  6) unknown  Item 3- Problems waking  1) none  2) mild  3) moderate  4) severe  5) very severe  6) unknown  Item 4- How SATISFIED/DISSATISFIED are you with your CURRENT sleep pattern?  1) very satisfied  2)satisfied  3) moderately satisfied  4) dissatisfied  5) very dissatisfied  6) unknown  Item 5- To what extent do you consider your sleep problems to INTERFERE with your daily functioning (e.g., daytime fatigue, mood, ability to function at work/daily chores, concentration, memory, mood etc.) CURRENTLY?  1) not at all  2) a little  3) somewhat  4) much  5) very much  6) unknown | Last two weeks | Variable was based on the mean score across items. Level 6 “unknown” was excluded from the mean estimations therefore the mean scores were calculated as the total score across all items divided by the number of items without “unknown” responses and missing responses. Higher mean scores reflect greater insomnia symptoms. An estimated 87.67% of the population answered a “known” value on all questions; 1.05% answered four questions; 4.96% answered three questions; 0.06% answered two questions; 0.03% answered one question; and 6.22% did not answer any of the questions (or responses “unknown.”. The mean scores were grouped into one of three groups. The "lower third" had a mean score of 1.0-2.6; the "middle third" included those with a mean score of 2.61-3.6. The “upper third" included participants who had a mean score of 3.61-5.0). |
| **Belongingness** | Items came from the Interpersonal Needs Questionnaire**.** Responses ranged from 1 (not at all true for me) to 7 (very true for me)  Item 1- These days, other people care about me.  Item 2- These days, I feel like I belong.  Item 3- These days, I feel that there are people I can turn to in times of need.  Item 4- These days, I am close to other people.  Item 5- These days, I have many supportive friends. | Current ("These days…") | Variable was based on the mean score across items. The mean is calculated as the total score across all items divided by the number of items answered. Higher mean scores reflect greater perceived belongingness. An estimated 94.65% of the population answered all questions; 2.07% answered four question; 0.38% answered three questions; 0.08% answered two questions; 0.00% answered only one question; 2.82% did not answer any of the questions. The mean scores were grouped into one of three groups.  The "lower third" had a mean score of 1.0-3.4; the "middle third" included those with a mean score of 3.5-5.5. The “upper third" included participants who had a mean score of 5.6-7.0. |

1. Details on the questions and response options come from the Military Suicide Research Consortium’s Common Data Elements Survey. Version 1.2.
